# Utilization of Citrus Peel Waste for Regulating Enzyme-Induced Carbonate Precipitation in Cement-Based Materials: Mechanical Performance and Freeze–Thaw Resistance

**DOI:** 10.3390/molecules31132308

**Published:** 2026-07-01

**Authors:** Yanzhi Meng, Xiang Su, Shujin Zhao, Qixiang Zan, Luyan Wang, Wenjuan Guo

**Affiliations:** School of Chemistry and Chemical Engineering, University of Jinan, Jinan 250022, China; 19906445534@163.com (Y.M.); sx126516@163.com (X.S.); zsj12561494@163.com (S.Z.); zqx1561456@163.com (Q.Z.)

**Keywords:** cement, urease, citrus peel, freeze–thaw cycles, mechanical

## Abstract

This study investigates citrus peel powder (CP) as an environmentally friendly admixture to regulate plant-derived urease (with soybean powder (SP) as the urease source) and to promote bio-mediated CaCO_3_ mineralization, thereby improving the mechanical and freeze–thaw (FT) resistance properties of cement-based materials. When CP is combined with urea and soybean urease, it exhibits a regulatory effect on urease activity. For the CPUD (CP-encapsulated urea combined with soy powder)-modified material with SP dosage in cement content of 0.2 wt%, the CP–urea modification treatment can effectively improve their mechanical properties and FT durability. The flexural and compressive strengths at 28 days are increased by 10.53% and 11.19%, respectively, compared to the blank group. After freeze–thaw cycles, the strengths are still 27.08% and 26.67% higher than those of the blank group, and their respective strength loss rates are 7.58% and −5.77% (negative indicating a net strength increase), compared with 21.31% and 9.48% for the blank group. X-ray diffraction, Fourier-transform infrared spectroscopy, and scanning electron microscopy analyses reveal that CP–urea promotes the formation and effective packing of calcium carbonate. Mechanistically, CP establishes a stable hydrogen-bonding network with both urea and urease, exerting a dual regulatory effect: it enhances the electrophilicity of urea while also creating a physical mass transfer barrier to precisely control biomineralization. Notably, CP can be directly used without pretreatment, offering a sustainable strategy for citrus peel waste valorization.

## 1. Introduction

Cement-based materials are the core materials in modern construction engineering [1], among which ordinary Portland cement (OPC) is widely used due to its large production scale and excellent cementitious properties [2]. However, their intrinsic limitations, such as low tensile strength, high brittleness, and easy cracking [3], make them vulnerable to the penetration of corrosive ions, which in turn accelerates various deterioration processes including carbonation, steel corrosion, and freeze–thaw (FT) damage [4]. Under cold climatic conditions, repeated freeze–thaw cycles (FTs) further accelerate crack propagation and the penetration of aggressive media [5,6], which seriously endangers the safety and service life of building structures [7]. As confirmed by Marsavina et al. [8], the failure of such materials is closely related to the initiation and propagation of cracks. Therefore, crack repair technologies for cement-based materials are of great significance for improving their FT resistance and durability, as well as extending their service life.

Furthermore, enzyme-induced calcite precipitation (EICP)-induced mineral precipitation acts as a strategic durability enhancement mechanism. Unlike atmospheric carbonation that compromises alkalinity, EICP facilitates the deposition of calcium carbonate within cracks and pores, creating a physical barrier that restricts the ingress of moisture, oxygen, and corrosive ions (e.g., Cl^−^, SO_4_^2−^) [9]. By densifying the microstructure and sealing transport pathways, this mineralization process helps maintain the protective alkaline environment around reinforcement, thereby mitigating corrosion risk and bolstering long-term structural integrity [10].

To date, EICP technology has been successfully applied in various fields such as soil improvement and building restoration [11]. For instance, Hua Yuan et al. [12] reported that before FT, the treated silt’s unconfined compressive strength and secant modulus rose 1.67 and 3 times, while porosity and permeability fell 20% and 93%; with more FTs and larger temperature swings, treated specimens retained lower porosity and better mechanical performance than untreated silt. In the field of concrete repair, the combination of chitosan and EICP has been shown to promote the formation of high-strength calcite crystals, which effectively reduces the permeability coefficient and mass loss of repaired tunnel lining cracks, providing an environmentally feasible solution for the repair of tunnel cracks.

Self-healing technology has emerged as an effective approach to reduce the deterioration of cement-based products and enhance their durability [13]. This technology enables autonomous crack repair and performance recovery by utilizing the intrinsic characteristics of materials or pre-embedded functional components, and it also offers the advantages of prolonged service life, cost savings, and improved efficiency [14]. Enzyme-induced carbonate precipitation (EICP) utilizes urease, a widely distributed enzyme in nature, as the core catalyst [15,16]. This technology promotes the decomposition of urea and generates carbonate ions in the alkaline environment of cement. These carbonate ions can then react with calcium ions in cement-based materials to form calcium carbonate precipitates, thereby achieving autonomous crack repair. A key advantage of EICP is that it directly uses plant-derived urease as the catalytic medium, which ensures low-cost acquisition and stable activity of urease. This characteristic endows EICP with strong operability, low overall cost, environmental friendliness, and favorable engineering controllability [17].

Nevertheless, slow urea decomposition restricts the practical engineering applications of crude-urease-based EICP. Specifically, while conventional high-purity commercial jack bean urease (e.g., Sigma-Aldrich) possesses a high labeled activity of 89,980 U/g [18], crude soybean urease typically yields a considerably lower specific activity, with the overall activity level ranging from 0.5 to 12.8 U/mL [19]. This significant efficiency gap restricts the rapid carbonate generation required for large-scale applications. Thus, the precise regulation of urease activity has become a key factor in expanding the application scope of EICP technology. Currently, researchers mainly adopt urease inhibition methods to control the hydrolysis rate; for example, N-(n-butyl) thiophosphoric triamide (NBPT) molecules can strongly inhibit urease by binding to its active sites [20], and this strategy has shown potential value in fields such as agriculture and biomedicine. However, using natural plant materials as media for urease activity regulation represents a brand-new research direction that has not yet been reported in existing studies.

Citrus fruits are the highest-yielding fruit category worldwide [21]. During processing, such as juicing and canning, the peel by-products account for 30–50% of the fresh fruit weight [22]. These by-products are mostly discarded directly, which not only causes resource waste but also brings environmental burdens. Citrus peels are rich in various bioactive components [23] and have great potential for high-value utilization, but their applications have long been concentrated in fields such as natural flavors and functional foods. For instance, Sharaf et al. [24] isolated hesperetin-7-rhamnoglucoside from citrus uranium fruit peel and demonstrated its urease inhibition activity through hydrogen-bonding interactions with active site residues, indicating the potential of citrus peel components to interact with urease. Compared with synthetic inhibitors such as NBPT, CP is low-cost, renewable, and environmentally friendly, while its diverse bioactive components enable versatile regulation of urease activity and add value to agricultural waste.

Based on this, citrus peel powder (CP) is introduced as an eco-friendly additive into the EICP system using soybean powder (SP) as the urease source. By constructing a CP-modified EICP system, precise regulation of plant-derived urease activity is achieved, leading to improved mechanical properties and FT durability of cement-based materials. The underlying modification mechanism of CP is also explored. This study presents a novel strategy for regulating urease activity through the use of CP, a natural plant waste that requires no pretreatment, enabling a low-carbon, low-cost, and environmentally friendly process. Moving beyond conventional regulation methods, this work not only offers a new perspective on enzyme activity control but also provides a practical pathway for the value-added reuse of citrus peel waste. Moreover, it contributes an innovative solution for the development of sustainable, high-performance cement-based repair materials.

## 2. Results and Discussion

### 2.1. Urease Activity

**Figure 1:**
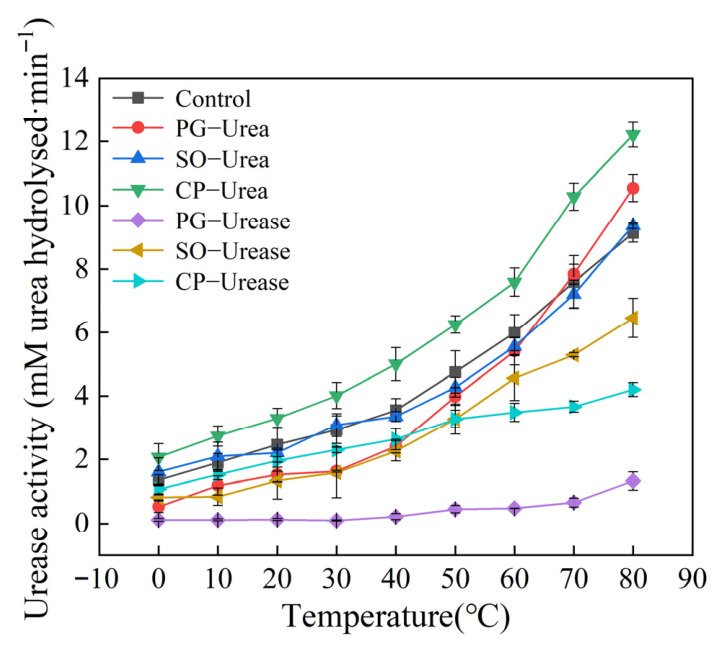
Effects of citrus peel powder (CP), high-ester citrus pectin (PG), and soybean polysaccharide (SO), applied at 2 wt% in aqueous dispersions, on the activity of soybean urease at various temperatures. Each encapsulating agent was used to pre-encapsulate either urea (CP–Urea) or urease (CP–Urease), and a non-encapsulated control group was included for comparison.

Figure 1 illustrates the effects of different encapsulating agents, including citrus peel powder (CP), high-ester citrus pectin (PG), and soybean polysaccharide (SO), on urease activity over the temperature range of 0–80 °C, with a non-encapsulated system as the control. Two encapsulation modes were employed, targeting either urea or urease, respectively, and their corresponding effects on urease-catalyzed urea hydrolysis were compared.

With increasing temperature, urease activities for all systems shown in Figure 1 display a pronounced and steady increase, indicative of accelerated urea hydrolysis. Notably, in the case of CP–urea, soybean urease shows pronounced temperature sensitivity between 40 and 80 °C, yet maintains high catalytic efficiency even above 70 °C. This thermal stability represents a key advantage of plant-based urease over microbial urease, which often fails at high temperatures [25,26].

Among the urea-encapsulated samples, CP–urea exhibits the strongest promoting effect, achieving a hydrolysis rate of 12.21 mM·min^−1^ at 80 °C—33.73% higher than that of the unencapsulated control—while PG–Urea shows the lowest activity. PG–Urea and SO–Urea primarily encapsulate urea via physical entrapment, reducing contact efficiency and suppressing hydrolysis. In contrast, CP–urea is rich in bioactive components (e.g., flavonoids and polysaccharides) that interact with urea through hydrogen bonding, improving its surface characteristics and enhancing urease accessibility. For urease-encapsulated samples such as CP–urease, the contact between urease and urea is reduced, leading to decreased activity.

Based on these results, CP–urea combined with SP was selected for incorporation into cement-based materials.

### 2.2. Mechanical Properties and Freeze–Thaw Resistance of Cement-Based Materials

#### 2.2.1. Flexural and Compressive Strength

**Figure 2:**
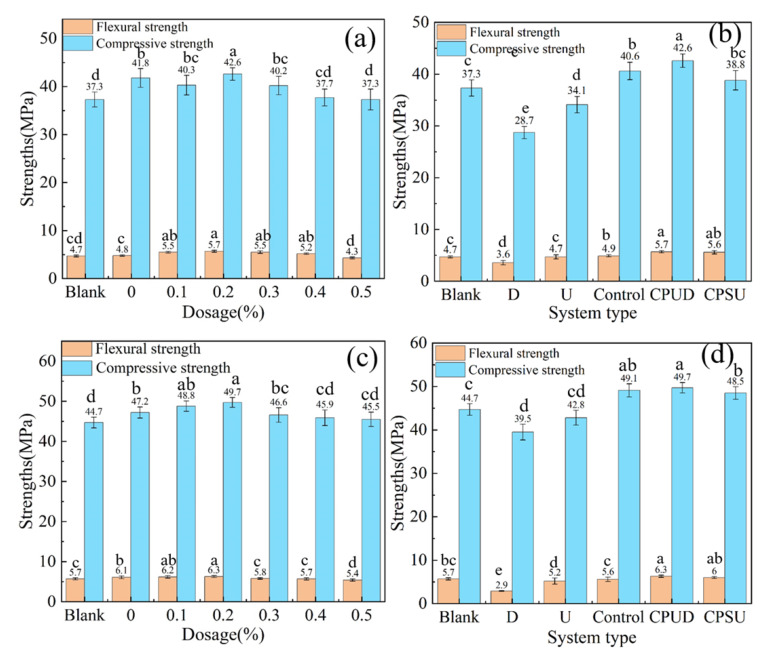
Flexural and compressive strengths of modified cement-based materials at 7 and 28 days. (**a**) Effect of soybean powder (SP) dosage on the 7-day strengths of CPUD-modified specimens. The x-axis denotes SP dosage as a mass percentage relative to the total cement; the “0 wt% SP” condition corresponds to specimens incorporating only citrus peel powder (CP) without SP or urea. (**b**) Comparative 7-day strength performance among specimens containing different admixture systems: Blank (plain cement paste, no admixtures), Control (0.2 wt% SP + urea), D (0.2 wt% SP only), U (urea only), CPUD (0.2 wt% SP + CP-encapsulated urea), and CPSU (CP-encapsulated SP (0.2 wt%) + urea). (**c**,**d**) Corresponding flexural and compressive strength values at 28 days. Different lowercase letters above bars within the same color (same mechanical index) indicate statistically significant differences evaluated by Tukey’s post hoc test (*p* < 0.05).

As illustrated in Figure 2, Figures S1 and S2, the x-axis represents the SP dosage expressed as a mass percentage of cement. For consistent data comparison, SP dosage was uniformly adopted as the independent variable in all plots. The reference systems (Blank, Control, U (urea only), and D (SP only)) were systematically compared with the CPUD (CP-encapsulated urea combined with soy powder) and CPSU (CP-encapsulated soy powder combined with urea)-modified systems, both designed to promote CP-mediated calcium carbonate precipitation, thereby enabling quantitative evaluation of the unique contribution of CP modification.

At both 7 and 28 days, all modified systems achieved peak mechanical performance at an SP dosage of 0.2 wt%.

- At 7 days, the CPUD group achieved flexural and compressive strength increases of 21.28% and 14.21%, respectively, while the CPSU group showed improvements of 19.15% and 4.02%. In the absence of SP, the addition of CP alone enhanced flexural strength by 2.13% and compressive strength by 12.06%;
- At 28 days, CPUD maintained superior performance with flexural and compressive gains of 10.53% and 11.19%, followed by CPSU (5.26% and 8.50%). Other systems (CP-only and Control) also exhibited improvements, though less pronounced than the CPUD and CPSU composites.

At higher SP dosages, the excessive incorporation of SP introduces abundant organic components that continuously adsorb onto cement mineral surfaces, thereby hindering sustained hydration (Figure S3). Furthermore, excess organic matter leads to heterogeneous biomineral precipitation and disrupts the continuous intergrowth of hydration products, a phenomenon widely validated in related EICP research. The synergistic action of these factors is ultimately responsible for the strength deterioration at later curing ages [27].

Data from Figure 2b,d further demonstrate that specimens modified solely with SP (Group D) or urea (Group U) exhibited inferior performance relative to the blank group, indicating that neither SP nor urea alone exerts a beneficial effect on mechanical properties. By contrast, the ternary CP–SP–urea system showed pronounced synergistic strengthening at an early age (within 7 days), with the CPUD group consistently attaining the highest strength among all samples.

Consequently, CPUD-modified specimens exhibit the highest flexural and compressive strengths among all tested samples at 28 days. This superior performance is likely associated with the optimization of biomineralization and matrix microstructure. The underlying microscopic mechanisms and comprehensive discussions regarding this enhancement are systematically presented in Section 2.3.2 and its subsequent sections.

This superior performance, which aligns with the enhanced urease activity observed in CPUD systems (Figure 1), underscores the critical role of CP in regulating the biomineralization process and densifying the cement matrix.

#### 2.2.2. Flexural and Compressive Strength of Cement-Based Materials Under Freeze–Thaw Cycles

**Figure 3:**
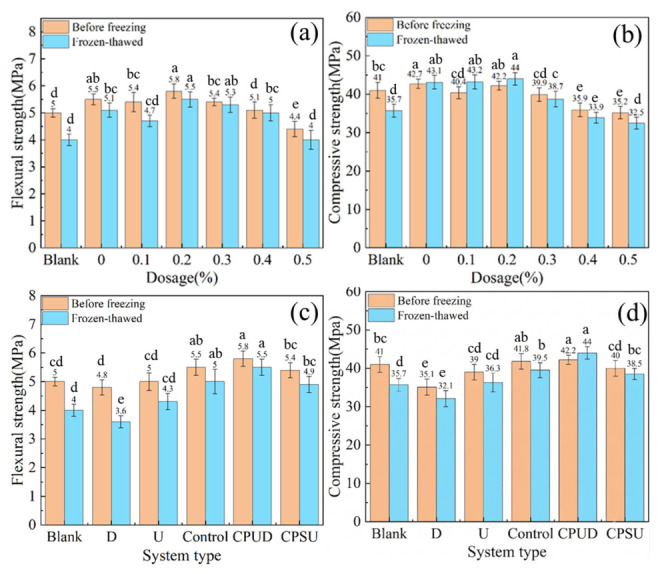
Flexural and compressive strength of CPUD-modified cement-based specimens (**a**,**b**) and comparative systems (**c**,**d**) after FTs following 7-day curing. The x-axis represents the soybean powder (SP) dosage as a mass percentage relative to cement. A total of 7-day strength comparisons are provided for: Blank (plain cement paste, no admixtures), Control (0.2 wt% SP + urea), D (0.2 wt% SP only), U (urea only), CPUD (0.2 wt% SP + CP-encapsulated urea), and CPSU (CP-encapsulated SP (0.2 wt%) + urea). Different lowercase letters above bars within the same color (same mechanical index) indicate statistically significant differences evaluated by Tukey’s post hoc test (*p* < 0.05).

Figure 3a,b demonstrate that, after 7 d of curing and subsequent FTs, the CPUD-modified specimens containing 0.2 wt% SP achieve peak flexural and compressive strengths, exceeding the unmodified blank group by 37.5% and 23.25%, respectively. The blank group exhibited flexural and compressive strength losses of 20.00% and 12.93%, respectively. At the optimal SP dosage, CPUD-modified materials incurred only 5.17% flexural loss and a net compressive strength gain of −4.27%, markedly outperforming the blank group and confirming exceptional microstructural resilience under cyclic freezing and thawing. Increasing the SP dosage beyond 0.2 wt% led to progressive reductions in both strengths. In contrast, the CP-only system showed robust freeze resistance, with flexural and compressive losses of 7.27% and −0.94%, respectively—both superior to the blank group.

As shown in Figure S4, CPSU-modified materials exhibited an overall strength reduction after freeze–thaw exposure. Nevertheless, at an SP dosage of 0.2 wt%, their flexural and compressive strengths were still 22.50% and 7.84% higher than those of the blank group, with corresponding strength loss ratios of only 9.26% and 3.75%—both lower than the blank group. These results confirm that the incorporation of CPSU effectively improves FT durability, though its enhancement is less pronounced compared with the CPUD-modified materials.

Figure 3c,d provide a quantitative comparison of the FT durability across all systems. The results indicate that the CPUD-modified materials exhibit superior frost resistance, as evidenced by the lowest flexural strength loss and the greatest compressive strength retention (manifested as a net gain) (Table 1) among all tested formulations.

**Figure 4:**
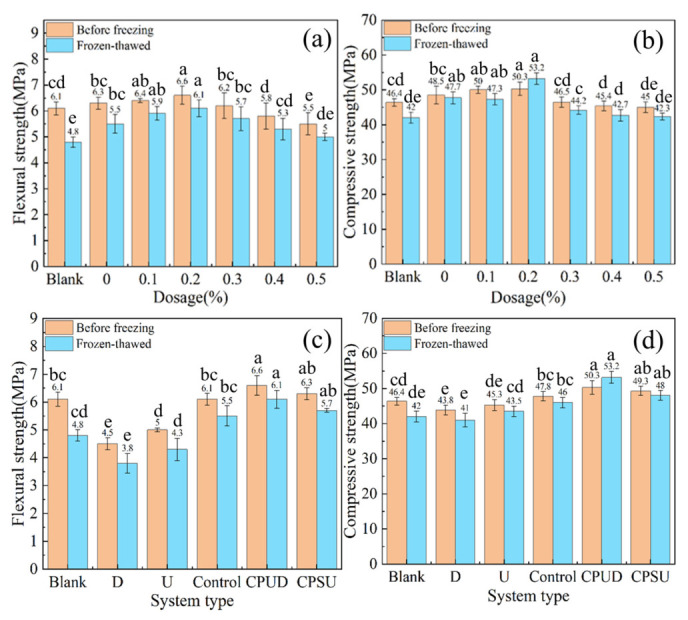
Flexural and compressive strength of CPUD-modified cement-based specimens (**a**,**b**) and comparative systems (**c**,**d**) after freeze–thaw cycles following 28-day curing. The x-axis represents the soybean powder (SP) dosage as a mass percentage relative to cement. A total of 28-day strength comparisons are provided for: Blank (plain cement paste, no admixtures), Control (0.2 wt% SP + urea), D (0.2 wt% SP only), U (urea only), CPUD (0.2 wt% SP + CP-encapsulated urea), and CPSU (CP-encapsulated SP (0.2 wt%) + urea). Different lowercase letters above bars within the same color (same mechanical index) indicate statistically significant differences evaluated by Tukey’s post hoc test (*p* < 0.05).

As depicted in Figure 4a–d, CPUD-modified specimens with a 0.2 wt% SP dosage achieved the maximum 28-day flexural and compressive strengths after FTs, exceeding those of the blank by 27.08% and 26.67%, respectively. Corresponding strength loss ratios were only 7.58% (flexural) and −5.77% (compressive), markedly lower than those of the blank (21.31% and 9.48%, respectively), confirming that CPUD incorporation significantly enhances the freeze resistance of cementitious materials. With increasing SP dosage (0.1–0.5 wt%), the flexural strength loss ratios were 7.81%, 7.58%, 8.06%, 8.62%, and 9.09%, while the compressive strength loss ratios were 5.40%, −5.77%, 4.95%, 5.95%, and 6.00%, respectively—the optimal FT durability is achieved with CPUD under the SP dosage of 0.2 wt%.

The CP-only modified specimens also exhibited improved freeze resistance, with flexural and compressive strength loss ratios of 12.70% and 1.65%, respectively. Consistent with this trend, both the control and CPSU-modified groups containing 0.2 wt% SP attained peak FT performance (Figures S5 and S6): flexural strength increased by 18.75% (CPSU) and 14.58% (Control), and compressive strength increased by 14.29% (CPSU) and 9.52% (Control), relative to the blank. Their post-FT strength loss ratios—flexural: 9.52% (CPSU) and 9.84% (Control); compressive: 2.64% (CPSU) and 3.77% (Control)—were consistently lower than those of the blank, with CPSU-modified materials outperforming Control across all metrics.

A systematic comparative analysis of the strength variations (Figure 4c,d) and strength loss ratios (Table 2) reveals that the CPUD-modified materials exhibit the most significant improvement in performance. Notably, this system achieves the lowest flexural strength loss and a net compressive strength gain after freeze–thaw exposure. This superior performance, ranking highest among all tested formulations, underscores the pronounced synergistic effect between CP and urea in stabilizing the cementitious microstructure and mitigating damage under cyclic freezing and thawing conditions.

#### 2.2.3. Freeze–Thaw-Induced Deterioration and Surface Morphology in Cement-Based Specimens

To further evaluate FT-induced deterioration and surface morphology, mass loss data after FTs (Figures S7 and S8) were analyzed, revealing no obvious mass loss but a slight mass gain due to water absorption [28]. However, this slight mass gain does not indicate an increased saturation susceptibility that would normally accelerate frost damage. Rather, as supported by the high strength retention and refined pore structure, the controlled mass evolution suggests that the dense matrix successfully restricted excessive water ingress, thereby effectively suppressing ice-expansion stress during freeze–thaw cycles.

**Figure 5:**
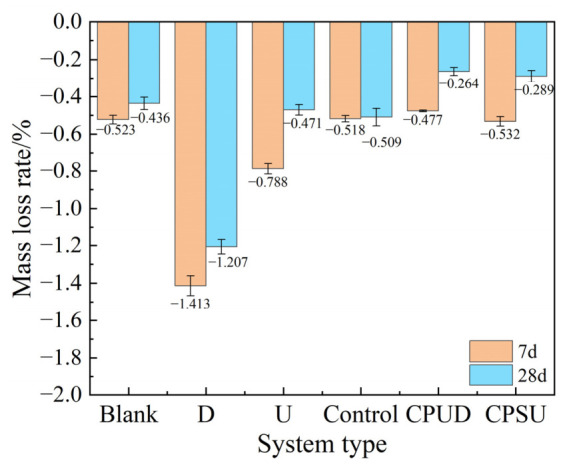
Mass loss ratios of modified cement-based specimens at 7 and 28 days, respectively, after freeze–thaw cycles. Blank (plain cement paste, no admixtures), Control (0.2 wt% SP + urea), D (0.2 wt% SP only), U (urea only), CPUD (0.2 wt% SP + CP-encapsulated urea), and CPSU (CP-encapsulated SP (0.2 wt%) + urea).

The results of the mass loss rate (reflecting the water absorption behavior during immersion) in Figure 5 show that with the extension of curing time, the values at 28 days are closer to zero than those at 7 days, indicating that the internal structure of the matrix is gradually densified with the progress of the hydration process. According to Equation (2), a negative mass loss rate explicitly signifies a net mass gain caused by water absorption. Therefore, a smaller absolute negative value (closer to zero) indicates less water absorption, demonstrating superior matrix impermeability. The 28-day mass loss ratios of the CPUD and CPSU-modified groups were significantly closer to zero, indicating their distinct advantages in inhibiting water absorption.

The 28-day water absorption rate of the CP-modified group was −0.307. CP is rich in natural polymer components, which can fill the internal pores of the matrix to a certain extent and make the internal structure denser, reducing water intrusion pathways, and thus effectively lowering the water absorption rate.

The surface morphology after FTs (Figures S9–S11) further validates the variations in durability among different systems: Figure S11 shows the D-modified group exhibits the most severe surface deterioration, characterized by extensive spalling and microcracks, consistent with its highest water absorption. The blank group shows obvious surface peeling and microcracks, while the U group displays numerous surface pores, with the Control group having relatively smaller pore sizes. In contrast, the CPUD and CPSU-modified groups retain intact surface structures without any visible FT damage. Their surfaces feature fewer and finer pores, in accordance with their lower water absorption. These results indicate that the CPUD and CPSU systems effectively refine the matrix pores, impede water ingress, reduce internal saturation, and mitigate frost heave stress, thereby substantially improving the freeze–thaw resistance of the materials.

#### 2.2.4. XRD Analysis

**Figure 6:**
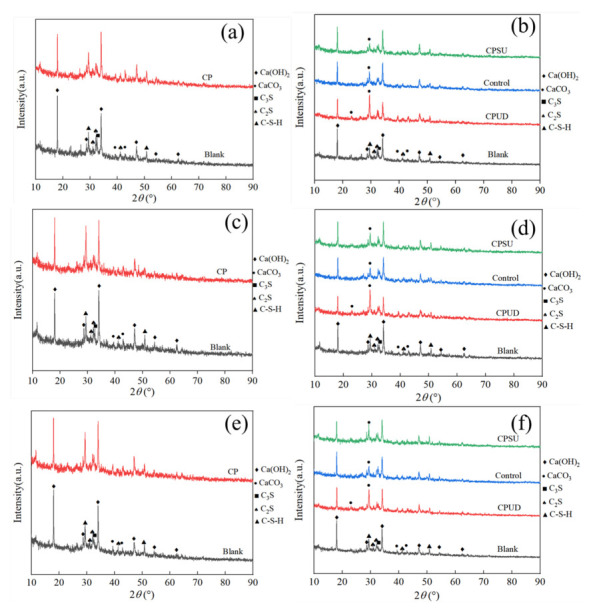
XRD patterns of modified cement-based materials at 28 days: (**a**,**b**) standard cured specimens, (**c**,**d**) freeze–thaw reference specimens, and (**e**,**f**) post freeze–thaw specimens. Samples: Blank, Control (0.2 wt% SP + urea), CP-doped, CPUD (0.2 wt% SP + CP-encapsulated urea)-modified, and CPSU (CP-encapsulated SP (0.2 wt%) + urea)-modified specimens.

XRD patterns reveal the characteristic broad hump of calcium silicate hydrate (C–S–H) centered at 2*θ* ≈ 29° (corresponding to the (110) reflection), along with weaker peaks at approximately 16.5°, 32.0°, and 49.8° [29]. As shown in Figure 6a,c,e, the blank specimen presents relatively weak C–S–H and calcite signals accompanied by intense diffraction peaks corresponding to unhydrated clinker phases (e.g., alite and belite), which is indicative of incomplete hydration. Specimens incorporating CP exhibit sharper and more intense C–S–H reflections—particularly at the (110) position—which suggests a more highly interconnected silicate network with improved structural polymerization (rather than crystalline growth, as C–S–H is inherently poorly crystalline). This effect is attributed to bioactive components in CP, including polysaccharides and organic acids, which chelate free Ca^2+^ ions via functional groups such as hydroxyl (–OH) and carboxyl (–COOH) moieties [30]. Supporting this mechanism, conductivity measurements (Figure S12) demonstrate that CP addition reduces the electrical conductivity of a 0.1 M CaCl_2_ solution by 10.08%, verifying its ability to effectively immobilize Ca^2+^ ions via complexation.

In cementitious systems, CaCO_3_ exists mainly as the calcite polymorph, characterized by a strong (104) reflection at 2*θ* = 29.1–29.7° and a (012) peak near 23.0° [31]. As illustrated in Figure 6b,d,f, the CPUD-modified system exhibits the most pronounced calcite (104) peak intensity compared to other groups; this result qualitatively indicates a more favorable environment for CaCO_3_ mineralization and tentatively suggests a synergistic enhancement effect of CP combined with urea on urease activity, which facilitates sustained and spatially uniform carbonate precipitation. Although both the Control and CPSU systems also facilitate calcite formation, their peak intensities are consistently lower than that of the CPUD-modified sample.

#### 2.2.5. FTIR Analysis for Cement-Based Specimens

**Figure 7:**
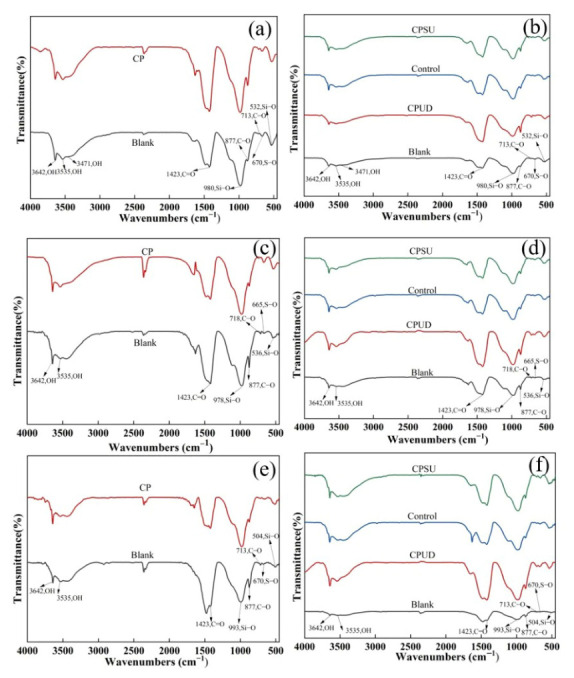
FTIR spectra of modified cement-based materials at 28 days: (**a**,**b**) standard cured specimens, (**c**,**d**) freeze–thaw reference specimens, and (**e**,**f**) post-freeze–thaw specimens. Samples: Blank, Control (0.2 wt% SP + urea), CP-doped, CPUD (0.2 wt% SP + CP-encapsulated urea)-modified, and CPSU (CP-encapsulated SP (0.2 wt%) + urea)-modified specimens.

As shown in the FTIR spectra in Figure 7, the absorption band near 3642 cm^−1^ is attributed to the O–H stretching vibration of portlandite (CH) formed during cement hydration [32]. The bands near 3521 cm^−1^ and 3446 cm^−1^ correspond to characteristic peaks of hydration products such as ettringite (AFt) and C–S–H. The asymmetric stretching vibration of C=O in carbonate appears near 1423 cm^−1^ [33]; bending vibrations of C–O in carbonate occur around 713 cm^−1^ and 872 cm^−1^; the Si–O stretching vibration in the range of 800–1200 cm^−1^ reflects the polymerized structure of C–S–H gel; the S–O bending vibration of SO_4_^2−^ in AFt is observed at 670 cm^−1^; and the bending mode of Si–O–Si appears between 450 and 538 cm^−1^.

From Figure 7a,c,e, the CP-modified materials show a larger peak area near 980 cm^−1^ than the blank. Acknowledging the overlapping nature of these bands with unhydrated phases and the semi-quantitative limitations of FTIR in cementitious systems, this enhanced peak area qualitatively reflects a higher degree of silicate polymerization and advanced structural ordering within the C–S–H gel network, which eventually contributes to the improved mechanical and frost-resistant properties. From Figure 7b,d,f, among the modified materials, CPUD exhibits the highest peak intensities for C–S–H, AFt, and carbonate bands, reflecting the strongest hydration and mineralization effects. CPUD not only promotes urea hydrolysis to form CaCO_3_ but also regulates Ca^2+^ availability, fostering the concurrent formation of AFt and C–S–H and enabling a synergistic generation of multiple hydration products—consistent with XRD observations.

#### 2.2.6. SEM Analysis

**Figure 8:**
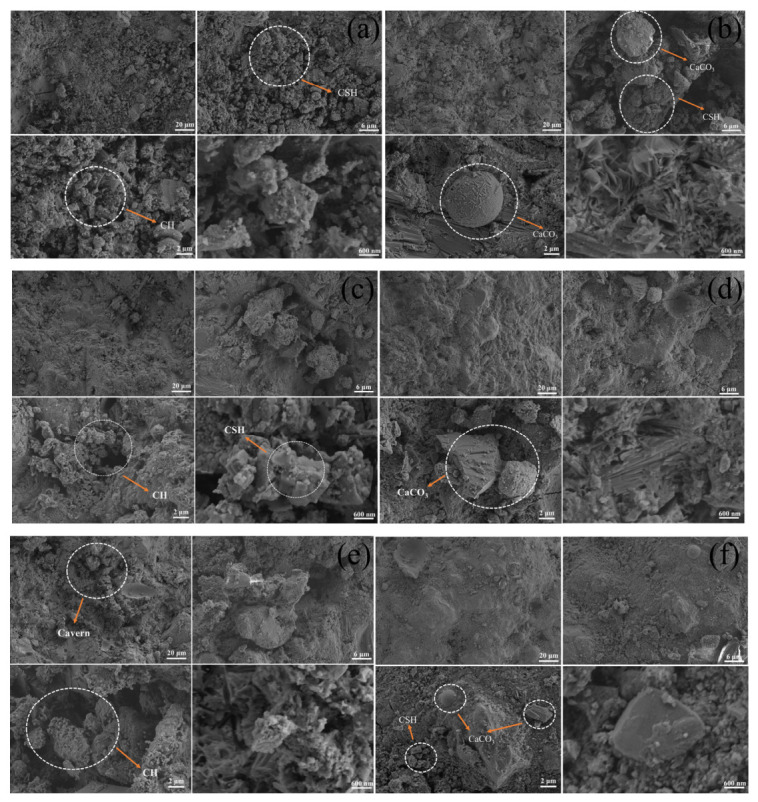
SEM images of Blank (**a**,**c**,**e**) and CPUD-modified (**b**,**d**,**f**) cement-based specimens at 28 days under standard curing, before and after freeze–thaw cycles.

To further investigate the microstructural evolution of blank and CPUD-modified cement-based specimens under FT conditions, SEM characterization was performed at 28 days under standard curing, before and after FTs (Figure 8). The hydration products, including CH, C–S–H, and CaCO_3_, were identified based on their typical morphological features: CH appears as plate-like or hexagonal crystals; C–S–H exhibits a fibrous or foil-like morphology; and CaCO_3_ is characterized by blocky, spherical, or rhombohedral crystalline particles.

As shown in Figure 8a,c,e, the blank specimens at 28 days of standard curing exhibit a typical hydrated cementitious microstructure. The hydration products, primarily consisting of CH crystals and C–S–H gels, construct a basic hardened framework that provides adequate structural integrity. After freeze–thaw cycles, the microstructure undergoes deterioration, accompanied by an increase in internal pores, indicating structural damage induced by the freeze–thaw action. For the CPUD-modified specimens (Figure 8b,d,f), a moderate densification of the matrix is observed at 28 days of standard curing, characterized by improved particle packing and reduced apparent porosity. The blocky CaCO_3_ crystals and the polymerized C–S–H gel network work synergistically to enhance interparticle cohesion. Even after suffering freeze–thaw cycles, the modified matrix maintains better microstructural uniformity, as the pore-filling effect of CaCO_3_ helps to effectively restrain the initiation and propagation of microdamage. Overall, these microstructural observations align objectively with the reported moderate improvements in mechanical performance, confirming that the enhancement of freeze–thaw durability is attributed to the synergistic effects of the interconnected hydration networks and the pore-filling action of CaCO_3_.

### 2.3. Mechanism Analysis

#### 2.3.1. Urease-Catalyzed Urea Hydrolysis Mechanism

**Figure 9:**
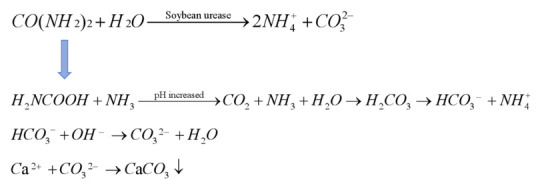
Urease hydrolysis reaction pathway schematic diagram.

**Figure 10:**
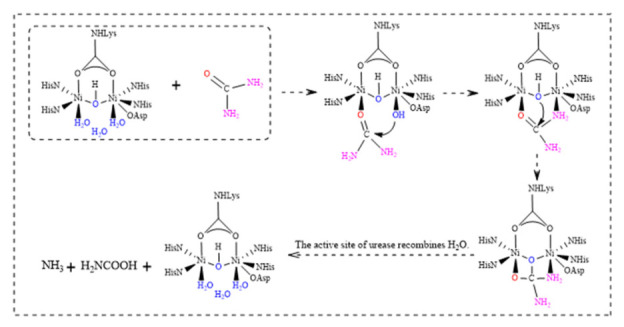
Schematic illustration of the urease-catalyzed urea hydrolysis mechanism.

Urease is a nickel-dependent metalloenzyme organized as a homohexamer, comprising six identical subunits. Each subunit contains a highly conserved catalytic center coordinated by two Ni^2+^ ions, which are ligated by histidine (His), aspartate (Asp), and lysine (Lys) residues. Upon substrate binding, urea coordinates to the dinickel center via its carbonyl oxygen atom, polarizing the carbonyl group and thereby increasing the electrophilicity of the carbonyl carbon [34]. This activation facilitates nucleophilic attack by a bridging hydroxide ion, leading to the formation of a tetrahedral carbamate intermediate [35,36]. Subsequent decomposition of this intermediate releases one molecule of ammonia and carbamic acid; the enzyme’s active site then re-binds a water molecule, and carbamic acid rapidly dissociates into a second molecule of ammonia and carbon dioxide. The CO_2_ then hydrates to form bicarbonate (HCO_3_^−^), while the accumulated NH_3_ protonates to yield ammonium ions (NH_4_^+^), resulting in localized pH elevation. In alkaline conditions, bicarbonate further equilibrates with carbonate ions (CO_3_^2−^) via deprotonation—a process illustrated schematically in Figure 9 and Figure 10.

CP is abundant in structurally diverse bioactive phytochemicals. The essential oil fraction is dominated by D-limonene (DL), a cyclic monoterpene representing the primary volatile component [37]. Its molecular structure is presented in Figure S13a. In addition to terpenoids, CP contains high levels of flavonoids, among which hesperidin is the most abundant and important constituent [38]. As a dihydroflavone O-glycoside, hesperidin possesses a dihydroflavone backbone decorated with multiple phenolic hydroxyl groups (Figure S13b). These structural moieties endow it with strong antioxidant activity and enable hydrogen bonding interactions that govern its aqueous solubility. Furthermore, low-molecular-weight organic acids, typified by citric acid, contribute to the weakly acidic nature of CP extracts. Citric acid contains three carboxylic acid groups, as shown in Figure S13c. The polysaccharide fraction of CP mainly consists of pectic polysaccharides, including homogalacturonan (HG), arabinan (Ara), and rhamnogalacturonan-I (RG-I) [39]. HG features a linear backbone composed of α-1,4-linked D-galacturonic acid (GalA) residues, the structural schematic of which is depicted in Figure S13d. The abundant hydroxyl, carboxyl, and hydrophobic groups in these components enable noncovalent interactions with urea and urease, thereby regulating urease activity and urea hydrolysis in the cementitious system.

#### 2.3.2. FTIR Analysis for Different Admixtures

**Figure 11:**
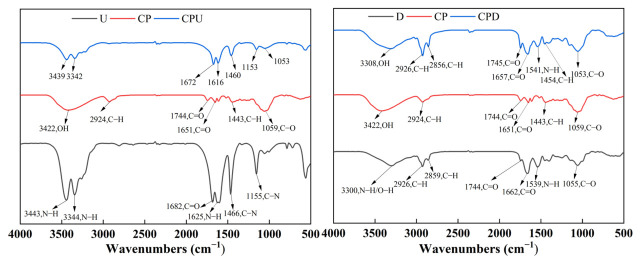
Comparative FTIR spectra of different admixtures. CP (citrus peel powder), CPU (CP–urea), and U (urea). CPD (CP–soybean powder (SP) complex), and D (SP).

FTIR was used to analyze the intermolecular interactions between CP and urea, and between CP and SP. In Figure 11, CP, CPU, U, CPD, and D correspond to citrus peel powder, the CP–urea complex, urea, the CP-SP complex, and SP, respectively.

Figure 11 reveals characteristic redshifts and broadening in key urea vibrational bands within the CPU system: the N–H stretching vibrations (3443 and 3344 cm^−1^ → 3439 and 3342 cm^−1^) exhibit pronounced broadening—indicative of O–H⋯N and N–H⋯O hydrogen bond formation [40]; the C=O stretching band shifts from 1682 to 1672 cm^−1^, a well-established spectroscopic signature of carbonyl involvement in hydrogen bonding [41], thereby increasing its dipole moment and bond polarity. Concurrently, the amino in-plane bending mode (1625 → 1616 cm^−1^) transitions from a sharp doublet to a broadened, unresolved feature. Notably, the intrinsic ester C=O stretch of CP at 1744 cm^−1^ also shifts to 1672 cm^−1^, strongly supporting the formation of C=O⋯H–N hydrogen bonds between CP’s carbonyl groups and the amino protons of urea [42]. This interaction further enhances the electrophilicity of the carbonyl carbon, rendering it more susceptible to nucleophilic attack by the urease-bound hydroxide. Additionally, the C–N stretching vibrations (1466 and 1155 → 1460 and 1153 cm^−1^) show consistent low-wavenumber shifts, reflecting diminished electron density and reduced bond order—direct consequences of hydrogen-bond-mediated electron withdrawal from the C–N bond.

Taken together, these spectral changes are consistent with the formation of a stable complex between CP and urea, primarily stabilized by intermolecular hydrogen bonding. Critically, this interaction appears to modulate the electron cloud distribution [43], inducing synergistic electronic effects: it augments the positive partial charge (δ^+^) on the urea carbonyl carbon—thereby heightening its electrophilicity—while simultaneously depleting electron density from the adjacent C–N bond. This correlation implies that the hydrogen-bonding-driven electronic modulation observed by FTIR could partially account for the kinetically favorable catalytic performance of the CP–urea system.

This correlation highlights the excellent catalytic performance of the CP-encapsulated urea system. In contrast, in the CP-encapsulated SP system, the presence of polyphenolic components in the encapsulation matrix may exert a significant influence on enzyme activity. Previous studies have confirmed that CP contains phenolic active substances [44], as evidenced by its FTIR spectrum (Figure 11), which exhibits a broad and intense hydroxyl absorption band in the range of 3200–3500 cm^−1^, corresponding to the stretching vibrations of phenolic hydroxyl groups in polyphenols. Polyphenols are well-known natural inhibitors of various enzymes, and their inhibitory activity arises from reversible noncovalent interactions—including hydrogen bonding and hydrophobic contacts—with enzyme active sites or allosteric pockets [45].

As illustrated in Figure 11, the broadened and red-shifted absorption centered at 3308 cm^−1^ in the CPD system originates from overlapping and perturbed N–H (from SP) and O–H (from CP phenolics) stretching vibrations. The magnitude of the redshift and peak broadening provides robust spectroscopic evidence for enhanced hydrogen-bond population and/or strength—specifically confirming O–H⋯N hydrogen bond formation [46]. Furthermore, the amide I band of D (1662 cm^−1^) shifts to 1657 cm^−1^ and overlaps with the flavonoid C=O absorption, which may indicate C=O⋯H–O hydrogen bonding between the peptide backbone carbonyl oxygen and phenolic hydroxyl protons. This interaction may sterically or electrostatically modulate urease–substrate recognition—for instance, by partially shielding negatively charged surface domains on SP that could otherwise interfere with urease binding or conformational dynamics.

Importantly, the FTIR spectrum of CPD retains all diagnostic peaks of both constituents: the ester C=O stretch of SP at 1745 cm^−1^, its aliphatic C–H stretches at ~2926 cm^−1^, and the C–O stretching vibration of CP at 1053 cm^−1^. Crucially, no new absorption features emerge—particularly in the regions diagnostic of peptide bond cleavage (e.g., no broadened carboxylate stretch near 1710 cm^−1^ or free amino stretch near 1550 cm^−1^) or oxidative degradation (e.g., carbonyl overoxidation or quinone formation). This confirms that the CP-SP interaction is purely physical and non-destructive, preserving the structural integrity of both macromolecular frameworks.

In summary, FTIR data demonstrate that multifaceted hydrogen-bonding networks between CP and SP systematically perturb the vibrational signatures of critical functional groups—including amino, carbonyl, and hydroxyl moieties. Based on this indirect evidence, we propose that these subtle modifications to the protein surface physicochemical landscape may represent a plausible molecular mechanism by which the CP-SP composite regulates urease enzymatic activity and fine-tunes urea hydrolysis kinetics. This further confirms the non-destructive physical encapsulation effect of CP on SP, and it is this effect that gives the CPSU-modified group better strength performance than the control group after 28 days of FTs.

Through comparative analysis of the two composite systems, the reason for the performance difference between the CPUD and CPSU groups can be clearly explained. In the CPUD-modified sample, CP mainly interacts with urea through hydrogen bonding, increasing urea electrophilicity and lowering hydrolysis activation energy, thereby promoting efficient urea decomposition by urease and supporting sufficient CaCO_3_ precipitation for pore filling in the cement matrix. At the same time, the weak physical hydrogen-bonding interaction between CP and SP mildly regulates urease activity without severe inhibition, ensuring a sustained and moderate hydrolysis rate. By contrast, in the CPSU-modified system, CP preferentially interacts with SP (urease), which partially inhibits enzyme activity and reduces the overall efficiency of urea decomposition. Although CPSU still improves cement performance, its weaker urea hydrolysis kinetics lead to lower CaCO_3_ formation and less effective microstructure densification. This mechanistic difference directly explains why CPUD exhibits the best overall performance in cement-based materials, while CPSU is slightly inferior.

#### 2.3.3. Zeta Potential and Particle Size Analysis

**Figure 12:**
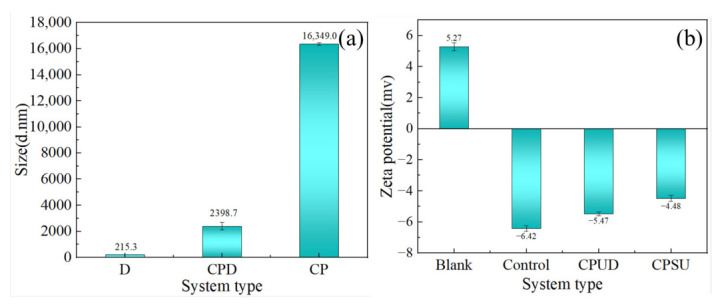
(**a**) Hydrodynamic particle sizes of D (SP, soybean powder), CP (citrus peel powder), and CPD (CP–SP complex) in aqueous dispersions; (**b**) zeta potentials of cement-based materials for different specimens, including the Blank, Control, CPUD and CPSU groups, respectively.

To investigate the particle size characteristics of individual components in aqueous dispersions and the surface electrochemical properties of cement-based materials under different modification systems, the particle sizes of D, CP, and CPD in aqueous dispersions, as well as the zeta potentials of specimens in different groups, were measured and analyzed, with the results presented in Figure 12.

Particle size data (Figure 12a) show that the CPD composite has a particle size of 2398.7 nm, which is considerably smaller than that of pure CP (16,349.0 nm). This remarkable result implies that SP particles adsorb onto the larger CP particles via heterogeneous aggregation. Such adsorption effectively disperses CP particles. The reduced particle size increases the specific surface area of the composite, thereby improving the exposure of active components in CP such as polyphenols. As a result, these components are more prone to interact with the active region of soybean urease, where they can occupy or regulate the enzyme active site through noncovalent interactions (including hydrogen bonds and hydrophobic effects, Figure 11), potentially contributing to the weakening of urease catalytic behavior. It should be noted that despite the pretreatment, the obtained apparent hydrodynamic diameter (e.g., approximately 16 μm for CP) approaches the upper reliable limit of classical DLS analysis, where the assumption of pure Brownian motion can be partially compromised by gravity-induced sedimentation and the irregular shapes of polydisperse particles. Therefore, rather than serving as an absolute geometric measurement, the DLS results in this study are utilized primarily as a qualitative and comparative indicator to evaluate the relative dispersion status and agglomeration trends of the particles in the suspension system.

By combining the results from particle size analysis, FTIR spectroscopy, and urease activity assays, it is plausible to infer that the blending of CP and SP facilitates the formation of a dense hydrogen bond network. This reduces the exposure of hydrophilic groups to the aqueous phase and enhances the apparent hydrophobicity of the composite [47]. Meanwhile, SP particles adsorb onto the CP surface via heterogeneous aggregation, forming a composite system with a smaller particle size. Together, the dense hydrogen bond network and the hydrophobic outer shell collectively form a mass transfer barrier, which significantly impedes the diffusion of urea molecules toward the urease active site and lowers substrate mass transfer efficiency, ultimately leading to the decline of urease activity.

As a key indicator of surface charge and dispersion stability in cementitious systems [48], zeta potential provides insight into the interaction behaviors related to urea hydrolysis and NH_4_^+^ generation under different modifications. The results can be explained as follows:

As shown in Figure 12b, the obtained zeta potential values are consistent with previous studies on cement systems [49]. The Blank group exhibits a positive zeta potential of 5.27 mV, consistent with the typical surface behavior of Portland cement in pore solutions where calcium ions (Ca^2+^) dominate the interfacial electrical double layer.

In the Control group (SP + urea), the negative shift in zeta potential indicates that the negatively charged groups on the soybean powder (SP) surface adsorb onto the cement particles. In the CPUD group, the hydrogen-bonding-mediated activation of urea facilitates efficient hydrolysis, producing NH_4_^+^, which may contribute to a slightly lower absolute zeta potential compared to the Control group. In the CPSU system, the interactions between CP and SP partially mask the negatively charged groups and urease active sites, resulting in the lowest absolute zeta potential.

Notably, all measured zeta potential values exhibit relatively low absolute magnitudes (approximately −5 to 6 mV), indicating that electrostatic repulsion is not the dominant factor governing system stability. Instead, the dispersion and stability of these modified systems are predominantly governed by steric hindrance. The adsorption of high-molecular-weight polysaccharides and flavonoids from CP onto the cement particles and SP–CP complexes creates a physical barrier, which is more dominant than electrostatic forces in the high-ionic-strength environment of cement pore solutions. Therefore, the variations in zeta potential observed across different groups reflect changes in the surface adsorption state and chemical environment, rather than simple fluctuations in electrostatic stabilization.

Such particle agglomeration and interfacial charge regulation optimize the particle packing structure within the cement paste, thereby enabling efficient pore filling in the cement matrix.

**Figure 13:**
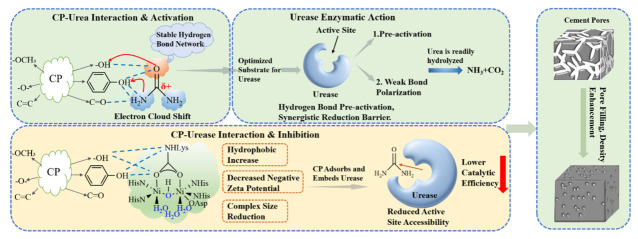
Mechanistic illustration of citrus peel powder (CP)-regulated urease activity and urea hydrolysis.

Figure 13 illustrates two competing mechanisms of CP in the CP–urea–urease–cement system and their effects on matrix pore densification. On one hand, the abundant polar functional groups on CP surfaces form stable hydrogen-bonding networks with urea. This pre-activation process optimizes the performance of urea as a urease substrate and accelerates its decomposition into NH_3_ and CO_2_, thereby promoting pore-filling carbonate mineralization. On the other hand, CP can directly interact with urease through hydrogen bonding, altering the zeta potential and particle size of the urease complex. The adsorbed CP reduces substrate accessibility and weakens the catalytic hydrolysis efficiency.

#### 2.3.4. UV–Vis Absorption Spectroscopy Analysis

**Figure 14:**
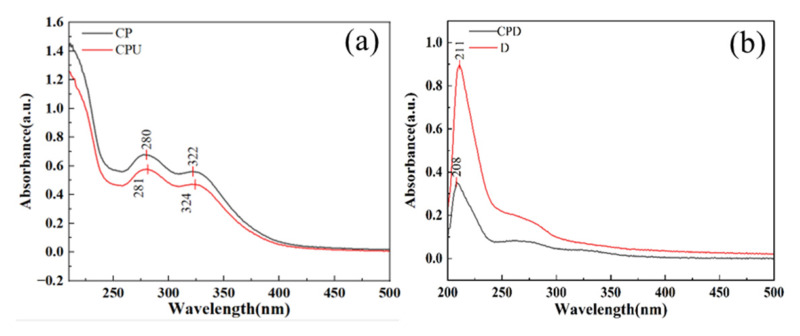
UV–Vis spectra of (**a**) CP (citrus peel powder) and CPU (CP–urea), and (**b**) D (SP, soybean powder) and CPD (CP–SP complex).

To reveal the intermolecular interactions and microenvironmental variations in different composite systems, UV–Vis spectroscopic analysis was performed, and the results are presented in Figure 14. UV absorption intensity and peak position are sensitive to changes in molecular structure or perturbations in the microenvironment [50]. The absorption peaks of CP at 280 nm and 322 nm correspond to the π→π* electronic transition of phenolic hydroxyl groups and the conjugated double-bond transition in flavonoids, respectively [51,52]. For the CPU composite, these peaks undergo a redshift to 281 nm and 324 nm, accompanied by significantly decreased intensity. Such shifts and attenuation arise from intermolecular hydrogen bonding between the phenolic and conjugated systems of CP and the C=O/–NH_2_ groups of urea, which may reduce the probability of valence-electron transitions.

D group (SP) exhibits a sharp and intense absorption peak at 211 nm, characteristic of the peptide bond moiety in soybean protein. In CPD, this peak intensity is significantly weakened due to the interactions between CP and SP [53]. Active components in CP form specific hydrogen bonds with urease through polar groups, while their hydrophobic moieties induce local desolvation at the binding interface, collectively generating a low-polarity microenvironment. This widens the energy gap for the π→π* transition of the peptide bond, leading to a blueshift in the UV spectrum [54,55]. The combined effects of hydrogen bonding and local desolvation might alter the conformation of key catalytic residues or the proton-transfer network within the urease active site, thereby impeding the access and binding of urea. Based on this indirect spectroscopic evidence, we propose that these effects may contribute to the reduced catalytic activity.

## 3. Materials and Methods

### 3.1. Raw Materials

In this study, P.O 42.5 OPC (Shandong Pingyin Shanshui Cement Co., Ltd., Jinan, China) was adopted. The chemical composition of the OPC was determined using a Wavelength Dispersive X-ray Fluorescence (WDXRF) spectrometer (Zetium, Malvern Panalytical, Almelo, The Netherlands). The analysis was conducted via the pressed-powder pellet method, and the detailed oxide compositions are presented in Table 3.

Tap water was used as mixing water for preparing cement pastes. Analytical-grade urea was supplied by Tianjin Damao Chemical Reagent Factory, Tianjin, China; soybean polysaccharide (SO) was purchased from Shandong Juyuan Biotechnology Co., Ltd., Yanggu, Liaocheng, China. Citrus peel powder (CP) was collected from Zhongtang Town, China; Soybean powder (SP) was supplied by Fengzhifang Agricultural Technology Co., Ltd., Linyi, China. Additionally, high-ester citrus pectin (PG, 100%) was purchased from DSM Finn Meiyi Pectin Yantai Co., Ltd., Yantai, China, and deionized water was also used.

### 3.2. Urease Extraction and Activity Test

Urease was extracted from plant-derived SP. Briefly, SP was mixed with deionized water at a set ratio, stirred magnetically for 30 min, and allowed to stand for 2–3 h. The supernatant was filtered through gauze and centrifuged at 3000 rpm for 30 min (repeated 2–3 times), and the clear liquid was collected after removing the surface lipid layer to obtain the crude urease solution. Two encapsulation systems were prepared using aqueous dispersions with 2 wt% CP: (i) CP-encapsulated soybean urease (CP–Urease) and (ii) CP-encapsulated urea solution (CP–Urea). Both systems were ultrasonicated for 20 min and then left to stand for 2 h prior to the activity assay. For the activity measurement, urea was subsequently added to the CP–urease system, while urease was subsequently added to the CP–urea system to initiate the hydrolysis reaction. Urease activity was determined using a conductivity-based kinetic assay. Specifically, the average conductivity increase over five consecutive 3 min intervals was calculated from real-time conductivity measurements during urea hydrolysis. Following Whiffin’s established protocol [56], a conductivity increase of 1 mS·cm^−1^·min^−1^ corresponds to a urea hydrolysis rate of 11.1 mmol·min^−1^. Enzyme activity was thus expressed as micromoles of urea hydrolyzed per minute (mM urea hydrolyzed·min^−1^).

### 3.3. Specimen Preparation

This experiment investigates the effects of different modification strategies on the mechanical properties of cement-based materials. Based on urease activity and activation energy tests, SP, CP, and urea powder were selected as raw materials, and two basic composite materials were designed: (i) urea powder encapsulated by CP, denoted as CPU, prepared by mixing CP with urea, ultrasonicated for 20 min, and then allowing the mixture to stand for 2 h; (ii) SP surface-modified by CP, denoted as CPS, prepared by mixing CP with SP, ultrasonicated for 20 min, and then allowing the mixture to stand for 2 h.

With the mass ratio of SP to urea powder fixed at 3:1, component adjustments were made to the two basic systems described above to obtain two corresponding ternary composite modifiers. Specifically, CPUD was prepared by introducing additional SP into the CPU system, while CPSU was obtained by adding extra urea powder into the CPS system. Subsequently, both CPUD and CPSU were incorporated into cement-based materials for comparative analysis with other reference systems.

All modified systems were evaluated against a blank group (without additives) and a binary control group in which SP and urea powder were directly added without encapsulation or modification. Meanwhile, a sample containing only SP (D) was prepared at a dosage of 0.2 wt% by cement mass, and the urea content (U) in cement was determined according to the 3:1 mass ratio of SP to urea based on this SP dosage.

(1) Preparation of composite modifiers: 2 wt% CP was first dispersed in deionized water and subjected to ultrasonication for 20 min, followed by 20 min of standing to facilitate the release of its active components. Subsequently, either urea powder or SP was introduced into the dispersion for surface modification. The mixture underwent a second ultrasonication treatment and was then allowed to stand for 2–3 h to ensure system stabilization. The as-prepared composite modifier was used for the fabrication of cement-based specimens.
(2) Cement-based specimen preparation: The cementitious mixtures were prepared with a constant cement mass of 1300 g and a fixed water-to-cement (w/c) mass ratio of 0.38 (494 g water). The stabilized composite modifier was prepared by introducing CP as a 2.0 wt% aqueous dispersion at a dosage of 2.0 wt% relative to the cement mass (26 g). To account for the water contribution from the CP dispersion, the total mixing water was adjusted to a net content of 468.52 g. Finally, the modifier was uniformly incorporated into the cement matrix alongside the designated amounts of SP and urea using a laboratory mortar mixer. The specific dosages of SP and urea for each mixture are detailed in Table 4. Mixing was performed in two stages: 2 min at low speed followed by 2 min at high speed, ensuring complete dispersion and homogeneity of the paste. The resulting fresh cement paste was then cast into prismatic molds (40 mm × 40 mm × 160 mm), vibrated briefly to remove entrapped air, and covered with polyethylene film to prevent moisture loss. Demolding was conducted after 24 h, and the specimens were subsequently cured under standard curing conditions (20 ± 1 °C, relative humidity ≥ 90%) until the designated testing ages.

(3) Mechanical testing: Flexural and compressive strength tests were carried out on cement-based specimens at prescribed curing ages in accordance with GB/T 17671-2021 [57]. The testing protocols specified in this standard are also widely applicable to cement paste systems [58]. All tests were performed using a computer-controlled universal testing machine equipped with dual flexural and compressive loading modules.

### 3.4. Setting Time Measurement

The initial and final setting times of cement pastes (w/c = 0.38) were determined using the Vicat apparatus in accordance with GB/T 1346-2011. Fresh pastes were cast into standard molds and kept under standard curing conditions. The initial setting time was defined as the duration from water addition until the Vicat needle failed to penetrate the paste to a depth of 4 ± 1 mm from the mold bottom. The final setting time was reached when the needle left no circular impression on the paste surface (penetration depth ≤ 0.5 mm). Results were reported as the average of three replicates.

### 3.5. Frost Resistance Test Procedure

Frost resistance was evaluated through FTs in accordance with the Standard for Test Methods of Basic Properties of Building Mortar (JGJ/T 70-2009) [59]. Each FT cycle comprised a freezing phase—conducted at −15 °C to −20 °C for 12 h—followed by a thawing phase in a water bath maintained at 15–20 °C for 12 h. Upon completion of the prescribed 5 FTs, specimens were subjected to mass measurement and mechanical testing to determine the mass loss ratio, flexural strength retention ratio, and compressive strength retention ratio—key indicators used to quantify frost resistance performance. The mass loss ratio and strength loss ratio were calculated using Equations (1) and (2), respectively.

Δ*f*_m_ = (*f*_m1 −_
*f*_m2_)/*f*_m1_ × 100 (1)

where Δ*f*_m_ represents the strength loss rate (%) after n FTs, *f*_m1_ is the average strength (MPa) of the reference specimen, and *f*_m2_ is the average strength (MPa) of the specimen after n FTs.

Δ*m*_m_ = (*m*_0_-*m*_n_)/*m*_0_ × 100 (2)

where Δ*m*_m_ represents the mass loss rate (%) after n FTs, *m*_0_ is the mass of the sample before the FTs test (g), and *m*_n_ is the mass of the sample after n FTs (g).

### 3.6. Hydration Characteristics and Microstructural Analysis

Phase composition and crystallinity were assessed via X-ray diffraction (XRD) using a Rigaku SmartLab SE diffractometer (Tokyo, Japan) [60]. Samples for XRD analysis were taken from the central portion of cement specimens after mechanical testing, immersed in anhydrous ethanol for 48 h, dried at 35 °C until constant weight, ground into powder, and sieved through a 100-mesh standard sieve. Diffraction patterns were acquired over a 2*θ* range of 10–90° at a scan rate of 10 °/min and a step size of 0.02°, with Cu Kα radiation (λ = 1.5418 Å).

Fourier-transform infrared (FTIR) spectroscopy was conducted on a Thermo Fisher Scientific Nicolet iS5 spectrometer (Thermo Fisher Scientific, Waltham, MA, USA) to characterize functional groups and identify major hydration products. The sample preparation procedure was the same as that for XRD analysis: the dried cement powder was thoroughly mixed and ground with potassium bromide (KBr) at a mass ratio of 1:100, and then pressed into transparent pellets for measurement. Spectra were recorded in transmission mode across the wavenumber range of 4000–500 cm^−1^, with a spectral resolution of 4 cm^−1^.

Microstructural morphology was examined using a Zeiss Gemini 300 field-emission scanning electron microscope (SEM, Carl Zeiss AG, Oberkochen, BW, Germany), operated at an accelerating voltage of 15 kV and working distance of 10 mm to evaluate the spatial distribution, morphology, and interfacial characteristics of hydration phases. After mechanical testing, cement specimens were cut and ground into small rectangular pieces. The areas intended for SEM observation were left unpolished to preserve the original microstructure, while the remaining surfaces were ground to smoothness. The specimens were then dried in an oven at 35 °C until constant weight, and subsequently attached to sample holders using conductive adhesive. Prior to observation, the specimens were gold-coated and subjected to vacuum treatment to ensure electrical conductivity.

### 3.7. Zeta Potential and Particle Size Measurements

The zeta potential of the cement suspension was determined using a Zetasizer Nano ZS90 instrument (Malvern Instruments Ltd., Malvern, Worcestershire, UK) [61]. The cement suspension was prepared with a water-to-cement mass ratio of 100:1. Prior to measurement, the suspension was homogenized by magnetic stirring for 5 min, followed by ultrasonic dispersion for 20 s, and then equilibrated at rest for 30 s to ensure uniform dispersion. Subsequently, the well-dispersed suspension was carefully injected into a zeta potential cuvette, which was then placed into the instrument for testing. All measurements were conducted at a constant temperature of 20 °C ± 2 °C to maintain test stability. Each sample was measured in triplicate, and the final zeta potential value was reported as the average of the three parallel readings to ensure data reliability.

The hydrodynamic particle size of each sample was measured via a dynamic light scattering (DLS) instrument. First, the sample was centrifuged at 4000 r/min for 3 min to remove large agglomerates. The middle layer of the supernatant was carefully collected to avoid interference from precipitates and floating impurities, and then further diluted 300-fold with deionized water to meet the instrument’s testing requirements. After proper dilution, the sample was transferred to the DLS instrument for particle size determination.

### 3.8. UV–Vis Absorption Spectroscopy

UV–Vis spectra were recorded using a TU-1901 double-beam UV–Vis spectrophotometer (Persee General Instrument Co., Ltd., Beijing, China). The scanning range was set from 200 nm to 500 nm, covering the characteristic absorption bands of phenolic compounds, flavonoid compounds in CP, and peptide bonds in soybean protein. To ensure test accuracy, the sample was diluted with 50 mM phosphate buffer (pH 7.0) to adjust the absorbance to the linear detection range of the instrument. Each sample was scanned three times continuously, and the average spectrum was taken as the final UV–Vis spectrum to reduce measurement errors.

### 3.9. Electrical Conductivity Measurement for Calcium Ion Complexation Assessment

The calcium ion (Ca^2+^) complexation capacity of CP was quantitatively evaluated via electrical conductivity measurements [62]. A 12.5 mg/mL aqueous CaCl_2_ solution was prepared, and CP was subsequently added under continuous stirring for 60 min. Conductivity was measured before and after the addition of CP using a calibrated Eutech PC 2700 conductivity meter (Thermo Fisher Scientific, Eutech Instruments Pte. Ltd., Singapore). All measurements were performed at 25.0 ± 0.2 °C, and triplicate readings were averaged to yield the final value. A decrease in conductivity relative to the blank CaCl_2_ solution was interpreted as indicative of Ca^2+^ immobilization through chelation or surface binding [63].

Data processing was performed using OriginPro 2022 (v9.9.0.225, OriginLab Corp., Northampton, MA, USA).

## 4. Conclusions and Foresight

In summary, natural plant waste CP can be employed as an eco-friendly admixture to regulate the catalytic activity of plant-sourced urease while simultaneously enhancing both the mechanical performance and freeze–thaw durability of cement-based materials. This work presents a systematic evaluation of CP’s efficacy in regulating the EICP process within cementitious systems, and the main findings are summarized below:

(1) CP encapsulating urea exhibited the highest urease activity at different temperatures. Significant improvement in mechanical properties and FT durability: For the CPUD-modified materials under SP dosage of 0.2 wt%, the CP-encapsulated urea powder modification treatment effectively improves the mechanical strength and durability. Compared to the blank group, the 28-day flexural and compressive strengths increased by 10.53% and 11.19%, respectively. After FTs, the modified specimens maintained flexural and compressive strengths 27.08% and 26.67% higher than those of the blank group, with significantly reduced strength loss rates (only 7.58% and –5.77%, compared to 21.31% and 9.48% for the blank group).
(2) Microstructural characterization by XRD, FTIR, and SEM confirmed that CPUD modification promotes uniform nucleation and dense deposition of calcium carbonate within the cementitious matrix. Further analyses using FTIR, particle size, and zeta potential measurements revealed that when CP encapsulates urea, a stable hydrogen-bonding network is formed between them, which facilitates noncovalent interactions and potential charge variations. The negative shift in zeta potential confirms the establishment of intermolecular interactions, thereby enhancing the intermolecular affinity within the system. In contrast, when CP-encapsulated SP is rich in urease, the absolute zeta potential decreases, indicating that CP binds to urease through hydrogen bonding and hydrophobic interactions, forming a stable complex on the enzyme surface. This complex hinders the diffusion of urea toward the active site. This encapsulation-targeted differential regulation enables CP to serve as a multifunctional admixture, providing effective regulation of the biomineralization process.

This study demonstrates that CP, an admixture derived from plant biomass waste, enables regulation of urease activity while significantly improving both the mechanical performance and freeze–thaw resistance of cement-based materials. These findings offer novel insights for the rational design of EICP-based cementitious systems and establish a sustainable pathway for the high-value utilization of agricultural residues in construction materials.

Finally, several limitations of this study should be acknowledged, including the relatively limited freeze–thaw testing program, the inherent limitations of the characterization techniques employed, and the assumptions underlying the proposed mechanistic interpretation. Future work will consult the classic reference *Properties of Concrete* by A.M. Neville [64] and incorporate stress–strain and load–deflection curves to further characterize the mechanical performance of the modified cement-based materials.

## Figures and Tables

**Table 1:** Variation in strength loss ratio of cement-based composites at 7 days.

System Type	Flexural Strength Loss Ratio/%	Compressive Strength Loss Ratio/%
Blank	20.00	12.93
D	25.00	8.55
U	14.00	6.92
Control	9.09	5.50
CPUD	5.17	−4.27
CPSU	9.26	3.75

**Table 2:** Variation in strength loss ratio of cement-based composites at 28 days.

System Type	Flexural Strength Loss Ratio/%	Compressive Strength Loss Ratio/%
Blank	21.31	9.48
D	15.56	6.39
U	14.00	3.97
Control	9.84	3.76
CPUD	7.58	−5.77
CPSU	9.52	2.64

**Table 3:** Chemical composition of ordinary Portland cement (OPC).

Components	Cement (wt%)
Loss of ignition (LOI)	5.61
Silicon dioxide (SiO_2_)	22.75
Aluminum oxide (Al_2_O_3_)	8.66
Ferric oxide (Fe_2_O_3_)	2.77
Calcium oxide (CaO)	51.54
Magnesium oxide (MgO)	4.91
Sulfur trioxide (SO_3_)	1.87

**Table 4:** Mix proportions of the investigated cementitious mixtures.

SP Dosage (wt%)	Cement (g)	SP (g)	Urea (g)
0.1	1300	1.30	0.43
0.2	1300	2.60	0.87
0.3	1300	3.90	1.30
0.4	1300	5.20	1.73
0.5	1300	6.50	2.17

## Data Availability

The data presented in this study are available in the article.

## Supplementary Materials

The following supporting information can be downloaded at https://www.mdpi.com/article/10.3390/molecules31132308/s1. Figure S1. Flexural and compressive strengths of modified cement-based materials at 7 days: the x-axis denotes soybean powder (SP) dosage as a mass percentage relative to the total cement; the “0 wt% SP” condition corresponds to specimens incorporating only citrus peel powder (CP) without SP or urea. (a) CPSU (CP-encapsulated SP + urea); (b) control (SP + urea). Figure S2. Flexural and compressive strengths of modified cement-based materials at 28 days: the x-axis denotes soybean powder (SP) dosage as a mass percentage relative to the total cement; the “0 wt% SP” condition corresponds to specimens incorporating only citrus peel powder (CP) without SP or urea. (a) CPSU (CP-encapsulated SP + urea); (b) control (SP + urea). Figure S3. Setting times of modified cement pastes: the x-axis denotes soybean powder (SP) dosage as a mass percentage relative to the total cement; the “0 wt% SP” condition corresponds to specimens incorporating only citrus peel powder (CP) without SP or urea. (a) Control (SP + urea); (b) CPUD (CP-encapsulated urea + SP); (c) CPSU (CP-encapsulated SP + urea). Figure S4. Variations in flexural and compressive strengths of 7-day CPSU (citrus peel powder (CP)-encapsulated soybean powder (SP) + urea)-modified cement-based materials under freeze–thaw cycles: the x-axis denotes SP dosage as a mass percentage relative to the total cement; the “0 wt% SP” condition corresponds to specimens incorporating only CP without SP or urea. Figure S5. Variations in flexural and compressive strengths of 28-day CPSU (citrus peel powder (CP)-encapsulated soybean powder (SP) + urea)-modified cement-based materials under freeze–thaw cycles: the x-axis denotes SP dosage as a mass percentage relative to the total cement; the “0 wt% SP” condition corresponds to specimens incorporating only CP without SP or urea. Figure S6. Variations in flexural and compressive strengths of 28-day control (soybean powder (SP) + urea)-modified cement-based materials under freeze–thaw cycles: the x-axis denotes SP dosage as a mass percentage relative to the total cement. Figure S7. Mass loss rates of 7-day CPUD (citrus peel powder (CP)-encapsulated urea + soybean powder (SP))- and CPSU (CP-encapsulated SP + urea)-modified cement-based materials after freeze–thaw cycles: the x-axis denotes SP dosage as a mass percentage relative to the total cement; the “0 wt% SP” condition corresponds to specimens incorporating only CP without SP or urea. Figure S8. Mass loss rates of 28-day CPUD (citrus peel powder (CP)-encapsulated urea + soybean powder (SP))-, CPSU (CP-encapsulated SP + urea)-, and control (SP + urea)-modified cement-based materials after freeze–thaw cycles: the x-axis denotes SP dosage as a mass percentage relative to the total cement; the “0 wt% SP” condition corresponds to specimens incorporating only CP without SP or urea. Figure S9. Surface of the cement specimen from 28-day CPUD (citrus peel powder (CP)-encapsulated urea + soybean powder (SP))-modified cement-based materials after freeze–thaw cycles. The mass percentage in the figure represents the SP content (relative to the total cement). Figure S10. Surface of the cement specimen from 28-day CPSU (citrus peel powder (CP)-encapsulated soybean powder (SP) + urea)-modified cement-based materials after freeze–thaw cycles. The mass percentage in the figure represents the SP content (relative to the total cement). Figure S11. Surface of the cement specimen from 28-day cement-based materials modified with different systems after freeze–thaw cycles: Blank (plain cement paste, no admixtures), Control (0.2 wt% soybean powder (SP) + urea), D (0.2 wt% SP only), U (urea only), CPUD (0.2 wt% SP + citrus peel powder (CP)-encapsulated urea), and CPSU (CP-encapsulated SP (0.2 wt%) + urea). Figure S12. Effect of citrus peel powder (CP) on the electrical conductivity of CaCl_2_ solution. Figure S13. Structural formulas of the main active components in citrus peel powder (CP): (a) D-Limonene; (b) Hesperidin; (c) Organic acids; (d) D-Galacturonic acid.
