# Supplementary material for: Utilization of Citrus Peel Waste for Regulating Enzyme-Induced Carbonate Precipitation in Cement-Based Materials: Mechanical Performance and Freeze–Thaw Resistance
Source: Molecules. 2026 Jul 1;31(13):2308. doi: 10.3390/molecules31132308 (PMC13362673; doi:10.3390/molecules31132308)
Supplement: Supplementary file 1 [file molecules-31-02308-s001.zip › molecules-4303953-supplementary.pdf]

Supplementary Material

Utilization of citrus peel waste for regulating enzyme-induced carbonate precipitation in cement-based materials: mechanical performance and freeze–thaw resistance

Yanzhi Meng, Xiang Su, Shujin Zhao, Qixiang Zan, Luyan Wang*, Wenjuan Guo*

School of Chemistry and Chemical Engineering, University of Jinan, Jinan 250022, China;

* Corresponding author. Email address: chm_wangly@ujn.edu.cn (L. Wang)

* Corresponding author. Email address: chm_guowj@ujn.edu.cn (W. Guo)

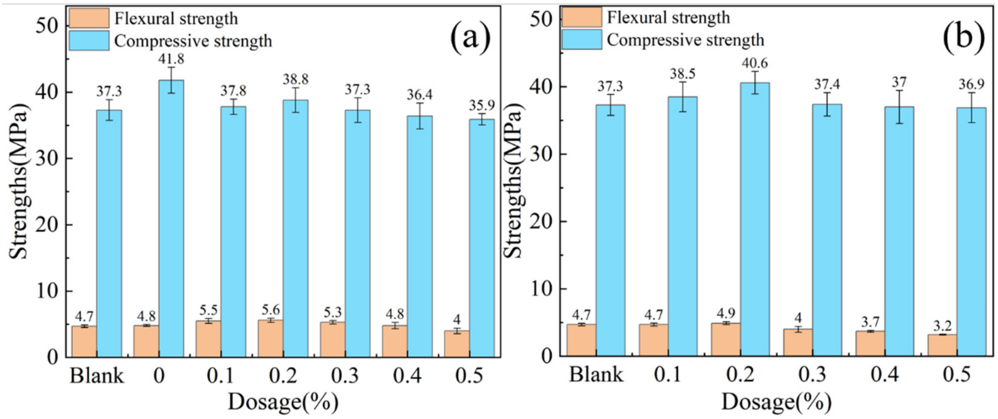

Figure S1. Flexural and compressive strengths of modified cement-based materials at 7 days: the x-axis denotes soybean powder (SP) dosage as a mass percentage relative to the total cement, the "0 wt% SP" condition corresponds to specimens incorporating only citrus peel powder (CP) without SP or urea. (a) CPSU (CP-encapsulated SP + urea); (b) Control (SP + urea).

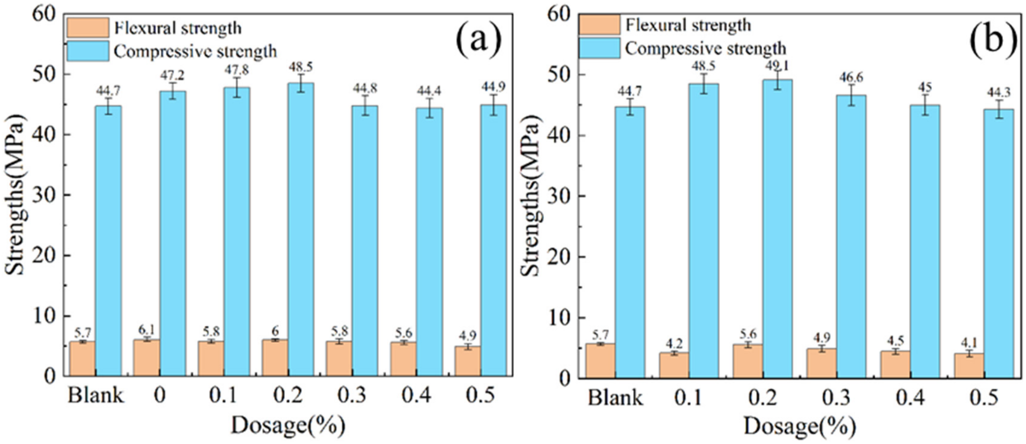

Figure S2. Flexural and compressive strengths of modified cement-based materials at 28 days: the x-axis denotes soybean powder (SP) dosage as a mass percentage relative to the total cement, the "0 wt% SP" condition corresponds to specimens incorporating only citrus peel powder (CP) without SP or urea. (a) CPSU (CP-encapsulated SP + urea); (b) Control (SP + urea).

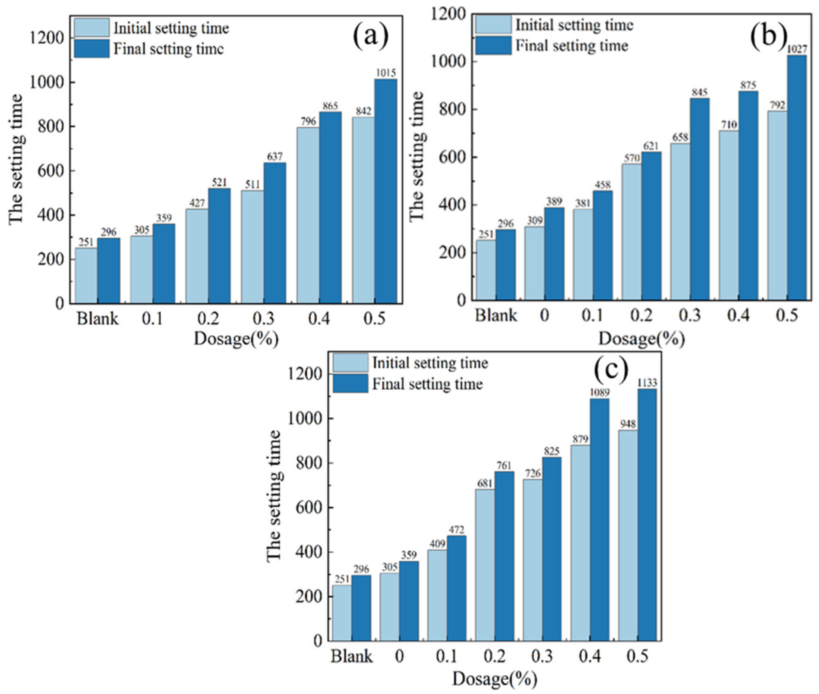

Figure S3. Setting times of modified cement pastes: the x-axis denotes soybean powder (SP) dosage as a mass percentage relative to the total cement, the "0 wt% SP" condition corresponds to specimens incorporating only citrus peel powder (CP) without SP or urea. (a) Control (SP + urea); (b) CPUD (CP-encapsulated urea + SP); (c) CPSU (CP-encapsulated SP + urea).

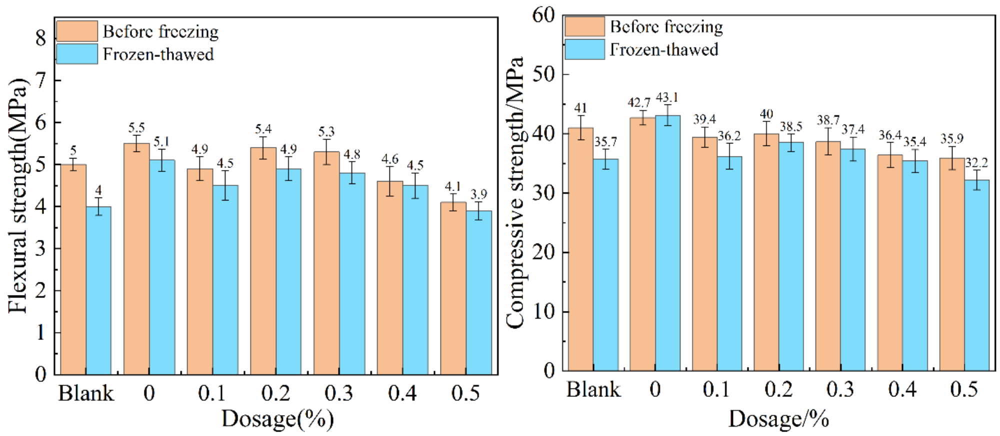

Figure S4. Variations in flexural and compressive strengths of 7 days CPSU (citrus peel powder (CP)-encapsulated soybean powder (SP) + urea) modified cement-based materials under freeze-thaw cycles, the x-axis denotes SP dosage as a mass percentage relative to the total cement, the "0 wt% SP" condition corresponds to specimens incorporating only CP without SP or urea.

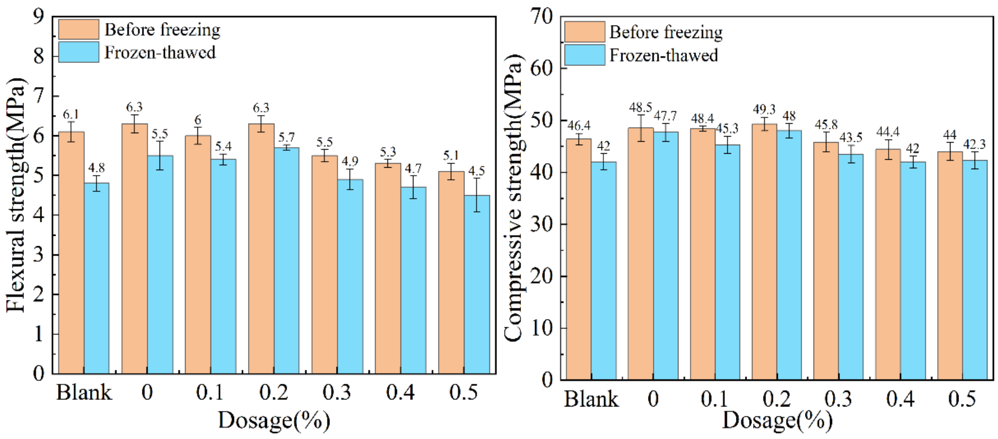

Figure S5. Variations in flexural and compressive strengths of 28 days CPSU (citrus peel powder (CP)-encapsulated soybean powder (SP) + urea) modified cement-based materials under freeze-thaw cycles, the x-axis denotes SP dosage as a mass percentage relative to the total cement, the “0 wt% SP” condition corresponds to specimens incorporating only CP without SP or urea.

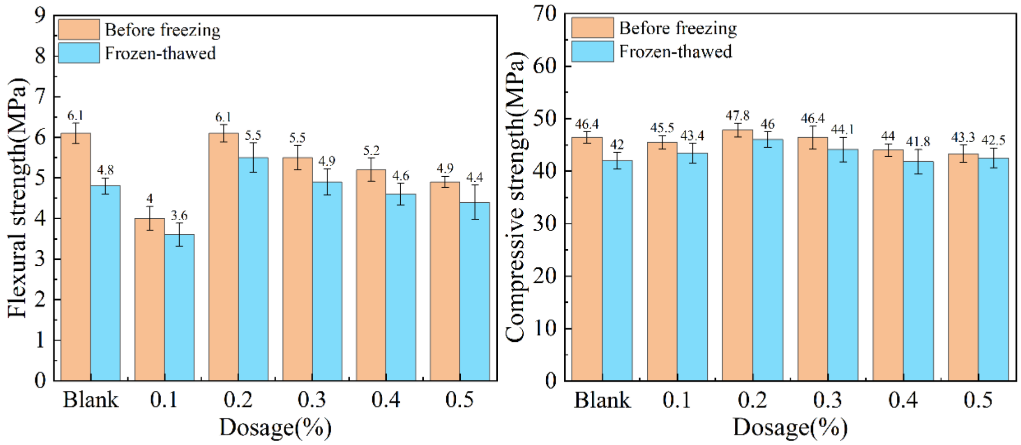

Figure S6. Variations in flexural and compressive strengths of 28 days Control (soybean powder (SP) + urea) modified cement-based materials under freeze-thaw cycles, the x-axis denotes SP dosage as a mass percentage relative to the total cement.

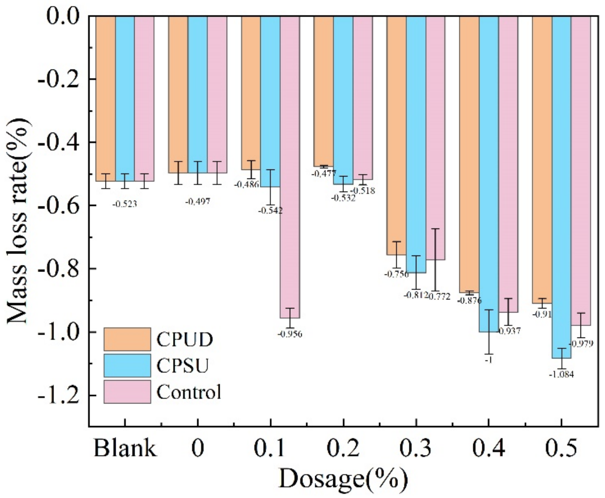

Figure S7. Mass loss rates of 7 days CPUD (citrus peel powder (CP)-encapsulated urea + soybean powder (SP)) and CPSU (CP-encapsulated SP + urea) modified cement-based materials after freeze-thaw cycles, the x-axis denotes SP dosage as a mass percentage relative to the total cement, the “0 wt% SP” condition corresponds to specimens incorporating only CP without SP or urea.

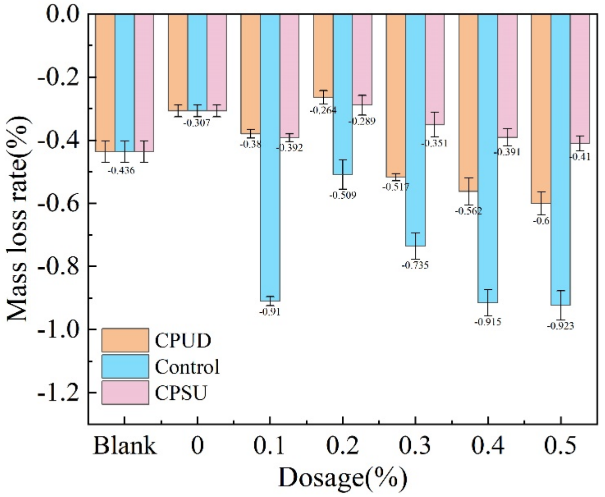

Figure S8. Mass loss rates of 28 days CPUD (citrus peel powder (CP)-encapsulated urea + soybean powder (SP)), CPSU (CP-encapsulated SP + urea), and Control (SP + urea) modified cement-based materials after freeze-thaw cycles, the x-axis denotes SP dosage as a mass percentage relative to the total cement, the “0 wt% SP” condition corresponds to specimens incorporating only CP without SP or urea.

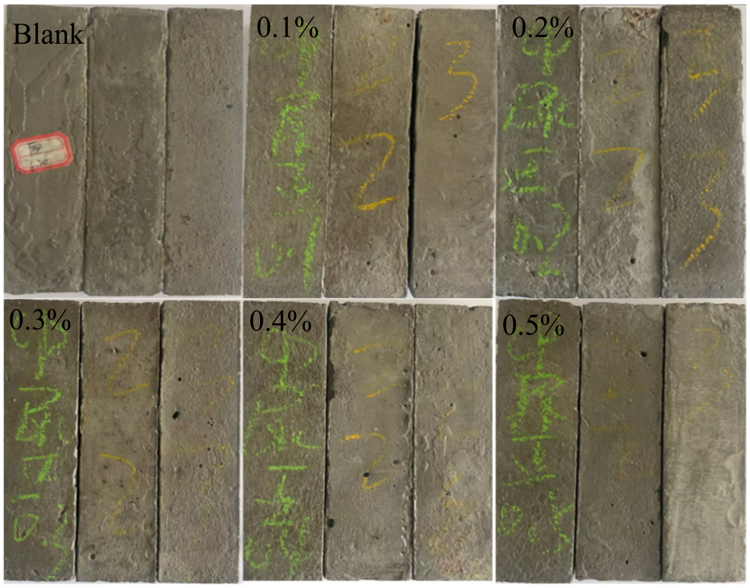

Figure S9. Surface of the cement specimen from 28 days CPUD (citrus peel powder (CP)-encapsulated urea + soybean powder (SP)) modified cement-based materials after freeze-thaw cycles. The mass percentage in the figure represents the SP content (relative to the total cement).

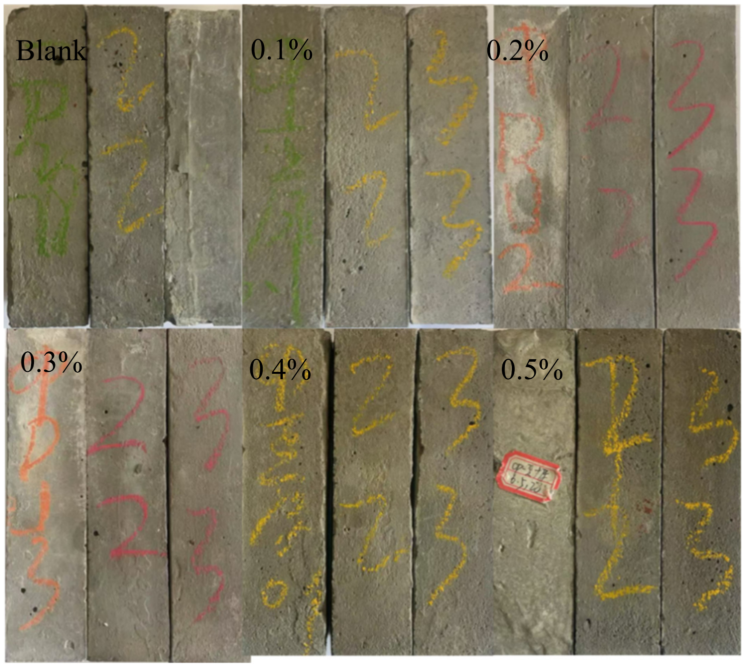

Figure S10. Surface of the cement specimen from 28 days CPSU (citrus peel powder (CP)-encapsulated soybean powder (SP) + urea) modified cement-based materials after freeze-thaw cycles. The mass percentage in the figure represents the SP content (relative to the total cement).

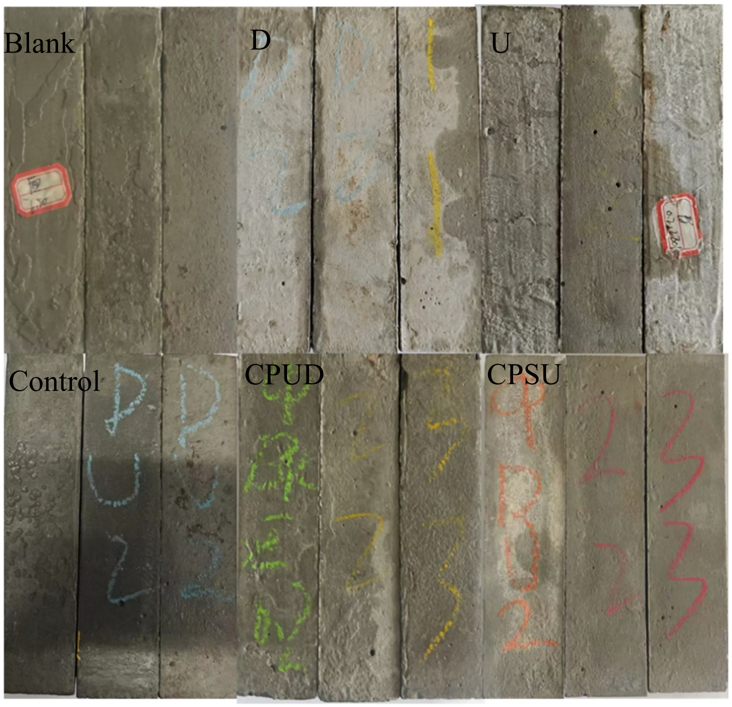

Figure S11. Surface of the cement specimen from 28 days cement-based materials modified with different systems after freeze-thaw cycles, Blank (plain cement paste, no admixtures), Control (0.2 wt% soybean powder (SP) + urea), D (0.2wt% SP only), U (urea only), CPUD (0.2 wt% SP + citrus peel powder (CP)-encapsulated urea), and CPSU (CP-encapsulated SP (0.2 wt%) + urea).

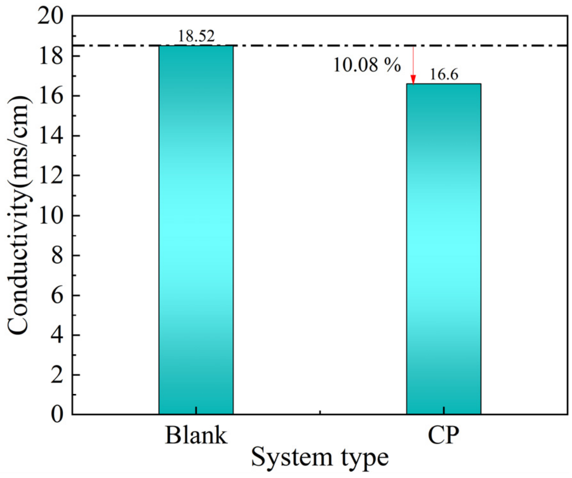

Figure S12. Effect of citrus peel powder (CP) on the electrical conductivity of $CaCl_2$ solution.

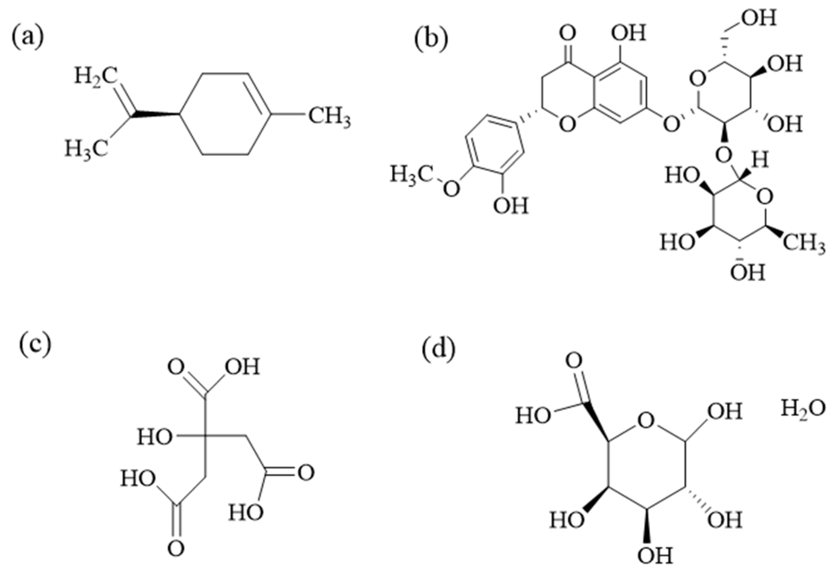

Figure S13. Structural formulas of the main active components in citrus peel powder (CP): (a) D-Limonene; (b) Hesperidin; (c) Organic acids; (d) D-Galacturonic acid.
